# Bone Engineering Scaffolds With Exosomes: A Promising Strategy for Bone Defects Repair

**DOI:** 10.3389/fbioe.2022.920378

**Published:** 2022-06-15

**Authors:** Mingming Zhang, Yi Li, Taojin Feng, Ran Li, Zhongqi Wang, Licheng Zhang, Pengbin Yin, Peifu Tang

**Affiliations:** ^1^ Department of Orthopedics, Chinese PLA General Hospital, Beijing, China; ^2^ National Clinical Research Center for Orthopedics, Sports Medicine and Rehabilitation, Beijing, China

**Keywords:** bone tissue engineering, scaffold, bone defect, bone regeneration, cell-free therapy, exosome

## Abstract

The treatment of bone defects is still an intractable clinical problem, despite the fact that numerous treatments are currently available. In recent decades, bone engineering scaffolds have become a promising tool to fill in the defect sites and remedy the deficiencies of bone grafts. By virtue of bone formation, vascular growth, and inflammation modulation, the combination of bone engineering scaffolds with cell-based and cell-free therapy is widely used in bone defect repair. As a key element of cell-free therapy, exosomes with bioactive molecules overcome the deficiencies of cell-based therapy and promote bone tissue regeneration via the potential of osteogenesis, angiogenesis, and inflammation modulation. Hence, this review aimed at overviewing the bone defect microenvironment and healing mechanism, summarizing current advances in bone engineering scaffolds and exosomes in bone defects to probe for future applications.

## Introduction

Bone is one of the important organs of the musculoskeletal system, which has load-bearing abilities and can perform locomotion as well as protect the internal organs. When suffering from high-energy trauma, nonunion, osteomyelitis, and tumor resection, loss of bone tissues will result in bone defects (Ma et al., 2021). Bone tissues are constantly remodeled and have better self-repair and regeneration ability, which allows the damaged bone tissues to fully recover to pre-injury integrity and mechanical properties (Majidinia et al., 2018). On the contrary, when the defects exceed the regeneration ability due to insufficient blood supply, local infection, drug side effects, malnutrition, etc., it will be difficult for large-sized bone defects to return to normal and seriously affect the patients’ motor function and life quality, which necessitates extra clinical treatments (Nauth et al., 2018).

For bone defects, the aim of rehabilitation is to recover the mechanical and functional integrity of the structure, so bone grafts and bone graft substitutes become suitable choices, which are widely explored for a better therapeutic effect (Li S. et al., 2021). The current available grafts include autologous bone grafts, allogeneic bone grafts, heterogenous bone grafts, and synthetic grafts, as well as cell-based therapy and cell-free therapy such as stem cells, bioactive factors, and extracellular vesicles (Baldwin et al., 2019; Wang and Yeung, 2017). Whether autologous bones or allogeneic and heterogenous bones, all have limitations for the treatment of bone defects, which make it difficult to meet the clinical demands (Schmidt, 2021). Consequently, it is urgent to develop alternative synthetic graft substitutes such as bone engineering scaffolds. An ideal bone engineering scaffold should meet the following criteria: excellent biocompatibility, biodegradability, osteoconduction, osteoinduction, and osteogenesis (Turnbull et al., 2018). To date, inorganic components, natural polymers, synthetic polymers, and metals, such as hydroxyapatite, collagen, poly(lactic acid), black phosphorus, and magnesium alloys, have been utilized in bone tissue engineering scaffolds (Amiryaghoubi et al., 2020; Bharadwaz and Jayasuriya, 2020; Zhang B. et al., 2021).

To fill in the bone defect sites and achieve desired therapeutic outcomes, bone engineering scaffolds are often integrated with stem cells, bioactive molecules, and extracellular vesicles (Brennan et al., 2020). Though bone engineering scaffolds provide stem cells with a platform for cell adhesion, migration, proliferation, and differentiation, the stem cells are also not ideal supplementary materials due to low survival rate, immunological rejection, tumorigenesis, and microthrombosis (Brennan et al., 2020). The extracellular vesicles, such as exosomes, have been proven to present parental cells and deliver bioactive molecules (e.g., nucleic acids, proteins, lipids, and metabolites), thus having the ability of osteogenesis, angiogenesis, and inflammation modulation, which promise to be desirable components combining with bone engineering scaffolds to repair bone defects (Huber et al., 2022; Li et al., 2020; Liu et al., 2018). The applications of exosomes and bone engineering scaffolds are still to be further researched, and there remain some problems to be solved. Therefore, this review will first focus on the bone defect microenvironment and bone healing mechanism. Based on this, we will discuss current treatments of bone defects and especially highlight bone engineering scaffolds and cell-free therapy. Then we will summarize the applications of exosomes and bone engineering scaffolds in bone defects. The potential problems and improvements to optimize exosome-integrated bone engineering scaffolds are also discussed.

## Bone Defect Microenvironment and Bone Healing Mechanism

The bone tissue structures are composed of cortical bone and cancellous bone (Buck and Dumanian, 2012). The cortical bone, consisting of osteons, acts as a supporter due to high mechanical strength. The cancellous bone, a porous structure, is composed of trabecular bones and bone marrows, which is the harbor of hematopoiesis and bone metabolism (Buck and Dumanian, 2012). In general, bone tissues are constantly in the state of dynamic absorption and remodeling, making it possible for bone tissues to adapt to growth, development, and dynamic mechanic load (Oftadeh et al., 2015). However, the normal function of the bone depends on its structural and compositional integrity, and bone regeneration depends on an ideal microenvironment. For a critical-sized defect, a bone engineering scaffold will provide the damaged bones with mechanical support and a microenvironment favorable for regeneration (Roseti et al., 2017). Therefore, an in-depth understanding of the bone defect microenvironment will provide clues for developing a better bone engineering scaffold system and promoting bone regeneration.

### Bone Defect Microenvironment

**FIGURE 1:**
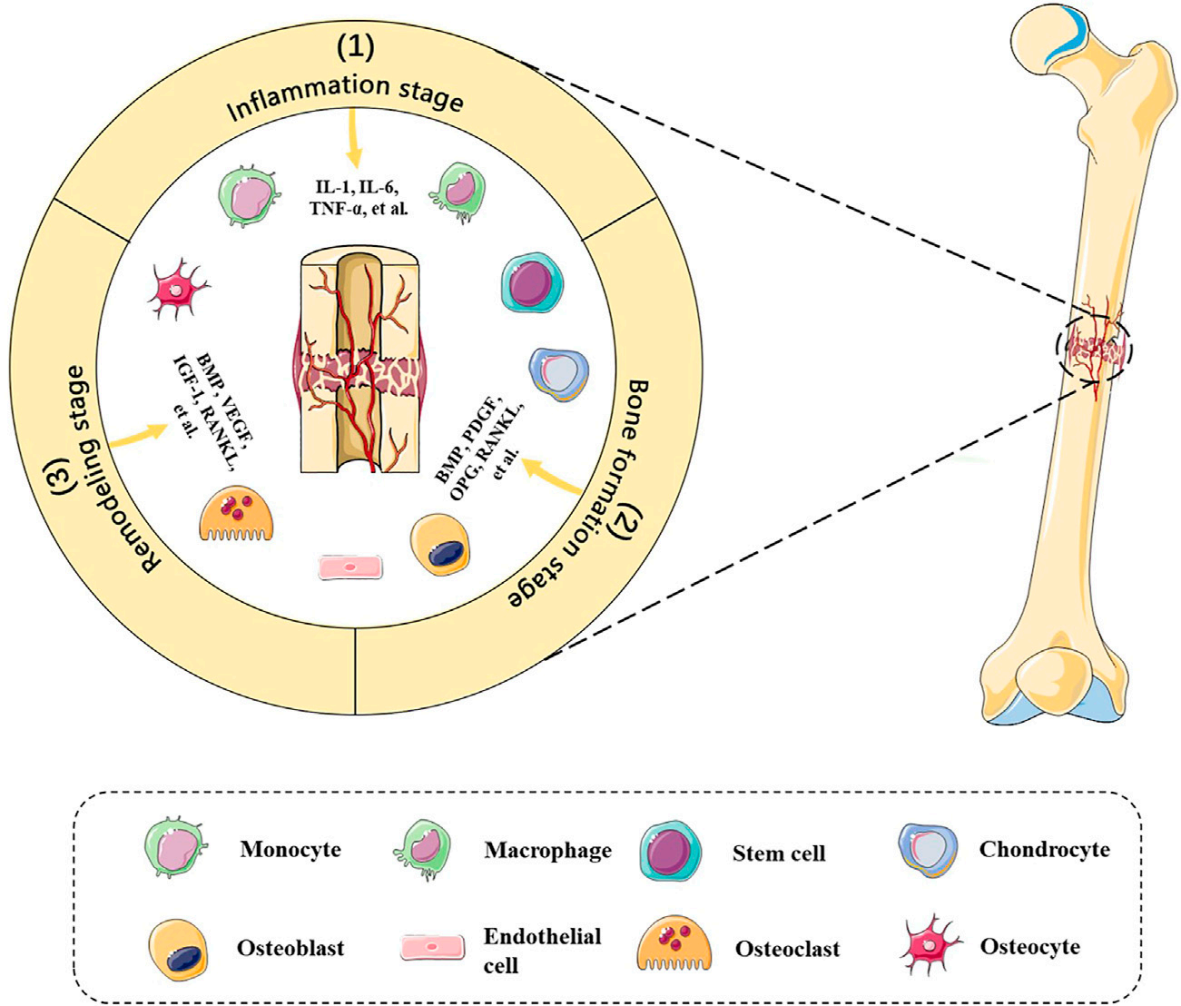
Bone defect microenvironment. Dynamic composition and cross-interactions of various cells and molecules are involved in the bone defect microenvironment. The bone healing stages include 1) inflammation stage, 2) bone formation stage, and 3) remodeling stage, which entails immune cells, chondrocytes, stem cells, osteoblasts, etc., and RANKL, PDGF, BMPs, TNF-α, etc., in a spatiotemporal manner.

The bone defect microenvironment refers to the dynamic composition and cross-interactions of various cells and molecules in the bone defect sites (Figure 1). The microenvironment is extremely complex. On one hand, it spans the various stages of bone healing in terms of time, including the inflammation stage, bone formation stage, and remodeling stage (Oryan et al., 2015). On the other hand, its composition includes a wide variety of cells, such as mesenchymal stem cells (MSCs), hematopoietic stem cells, immune cells, endothelial cells, osteoblasts and osteoclasts, and various bioactive factors, such as receptor activator of nuclear factor-kappa B ligand (RANKL), platelet-derived growth factor (PDGF), bone morphogenetic proteins (BMPs), and interferon-gamma (IFN-γ), which engage in osteogenesis, angiogenesis, and inflammation modulation (Safari et al., 2021; Zhou et al., 2021). Detailed summaries and discussions have been reviewed by Zhu et al. (2021). In the meanwhile, a recent single-cell sequencing study revealed that skeletal muscle-derived mesenchymal progenitors also engage in bone repair, which will explain why adjacent tissues also matter (Julien et al., 2021). In addition, Zhang H. et al. (2021) suggest that B cells are key regulators of bone healing in the bone marrow microenvironment due to the opposite pattern between B cells and bone formation and resorption activities. Considering the fact that outcomes of bone defect healing are uncertain under the influence of different risk factors, such as age, nutritional status, and contamination degree (Nicholson et al., 2021), we suspected that differences in the bone defect microenvironment may contribute to it. Therefore, further research studies on the bone defect microenvironment will help to explain the bone healing mechanism and pathogenesis of nonunion and delayed union, providing novel ideas and more personalized strategies for clinical practice.

### Bone Healing Mechanism

The defects sites initiate a cellular cascade to repair injury and promote regeneration shortly after the occurrence of bone defects. These cells participate in several continuous events, including hematoma formation, inflammatory reaction, fibrous callus formation, intramembranous ossification, endochondral ossification, and bone remodeling, which accompany an orderly cascade of anabolism and catabolism (Li et al., 2019). Specifically, blood clots in the damaged areas and immune cells migrate to remove the necrotic components. Next, recruited fibroblasts, osteoprogenitor cells, and MSCs proliferate and differentiate to form a fibrous tissue, followed by intramembranous and endochondral ossification. In the final stage, the new bone tissues are constantly absorbed and remodeled under mechanical forces, forming an orderly bone structure and returning to normal function (Kalfas, 2001; Zhu et al., 2021). Intramembranous ossification means that MSCs migrate and proliferate to form condensation, differentiate into osteoblasts, and secrete collagen, followed by vascular ingrowth and cortical bone and cancellous bone formation (Percival and Richtsmeier, 2013). Endochondral ossification means that MSCs differentiate into chondrocytes and secrete collagen matrix, followed by vascular ingrowth and cartilage degradation, finally forming the primary ossification center and secondary ossification center, and forming mature bone structure (Mackie et al., 2011). In addition, many other cytokines are also involved in the process of bone healing (Zhu et al., 2021). Although bone tissues have the remarkable ability of healing, bone defects still do not return to normal when the defect ranges exceed the critical-sized bone defect, which means loss of a length exceeding 2–2.5 times the diameter of the damaged bone (Wiese and Pape, 2010). This will overwhelm the ability of bone regeneration because of mechanical instability and biological disadvantage, indicated by poor vasculature, bone nonunion, and pseudarthrosis, which requires clinical treatments to support mechanical stability and a suitable microenvironment so as to achieve functional reconstruction (Elliott et al., 2016). Consequently, the in-depth understanding of bone biology, bone defect microenvironment, and bone healing mechanisms will provide references for the design and application of bone engineering scaffolds with bioactive factors.

## Current Treatments of Bone Defects

The interventions of large segmental bone defects usually require repair and reconstruction techniques (Nauth et al., 2018). However, the treatments of bone defects remain a striking challenge to date because of the shortage of autologous bone tissues and the lack of ideal graft materials (Wang and Yeung, 2017). With the development of materials science and engineering technology, the combination of advanced manufacturing technology with ideal materials, bone-implant interface modification, and the supplement of bioactive factors provides a broad space for the treatment of bone defects (Tovar et al., 2018; Turnbull et al., 2018).

### Bone Graft Reconstruction

Bone grafts include autologous bone grafts, allogeneic bone grafts, and heterogenous bone grafts, all of which have different characteristics (Shang et al., 2021). Autologous bone grafts have fresh cortical and cancellous bone tissues containing viable osteoblasts, osteocytes, MSCs, and growth factors, thus possessing good osteoconductive, osteoinductive, and osteogenic properties. Owing to the maintenance of osteogenic potential and basis, autologous bone grafts are considered as the gold standard in the treatment of bone defects (Baldwin et al., 2019). However, some shortcomings restrict its application, for example, limited availability, donor site infection, hematoma, and pain (Schmidt, 2021). Allogeneic bone grafts refer to bone tissues from other individuals, which are immunogenic and have the risk of the spread of potential pathogens (Wang and Yeung, 2017). Therefore, processed and modified allogeneic bones overcome their own shortcomings and become the most available grafts, considered the best alternative to autografts. Demineralized bone matrix (DBM) is a highly processed allogeneic graft, which is often used to fill in bone defects (Hao et al., 2022). Similarly, heterogenous bone grafts face the same concerns, such as immunogenicity and disease transference (Amiryaghoubi et al., 2020). In general, due to the aforementioned drawbacks, bone grafts may not be the optimal ones, so it is necessary to design and manufacture promising bone engineering grafts with excellent osteoconduction, osteoinduction, and osteogenesis.

### Bone Graft Substitute Reconstruction

The development of bone graft substitutes aimed at simulating natural bone tissues to produce bone scaffolds with excellent capacity for osteoconduction, osteoinduction, osteogenesis, and angiogenesis (Tan et al., 2021). At present, a variety of alternative materials have been utilized for scaffold engineering, including inorganic components (e.g., hydroxyapatite, CaP cement, and ceramics), natural polymers (e.g., collagen, chitosan, alginate, and hyaluronic acid), synthetic polymers (e.g., poly(lactic-co-glycolic acid), poly(lactic acid), and poly(caprolactone)), and metals (e.g., magnesium and magnesium alloys, Zn and Zn alloys, and titanium and titanium alloys) (Table 1) (Amiryaghoubi et al., 2020). The summaries of types and manufacturing technologies have been reviewed recently (Bharadwaz and Jayasuriya, 2020; Wang and Yeung, 2017). In addition, new engineering techniques are used to solve practical application problems, for example, a microfluidic 3D printing strategy fabricates photothermal responsive channeled scaffolds, which facilitate vascular ingrowth and bone regeneration (Wang et al., 2021). In general, advances in materials science and engineering technology gradually make it possible for more bone engineering scaffolds to be used in clinical practice.

**TABLE 1:** Brief comparison of bone engineering scaffold materials.

Material type	Example	Advantage	Disadvantage	References
Inorganic components	Hydroxyapatite, CaP cements, and ceramics	High compressive strength and low ductility	Brittleness	Gao et al. (2014)
Natural polymers	Collagen and chitosan	Good biocompatibility, osteoconductivity, and low immunogenicity	Degradation rate difficult to control and low mechanical stability	Ebhodaghe, (2021)
Synthetic polymers	Poly(lactic-co-glycolic acid) and poly(lactic acid)	Controlled degradation rate, the possibility to design or tune bone mechanical properties, plasticity, and the potential to deliver soluble molecules	Lower ability to interact with cells	Amiryaghoubi et al. (2020)
Metals	Magnesium alloys and titanium alloys	High strength and modulus, good biocompatibility	Degradation and hydrogen generation	Zhang et al. (2021c)
Ideal scaffold	Biocompatible, non-toxic, bioresorbable, biodegradable, non-immunogenic, bioactive, biomimetic, customized shape, high porosity, and mechanical properties			Roseti et al. (2017)

### Masquelet’s Induced Membrane Technique

Masquelet’s induced membrane technique is a two-stage surgical procedure to treat segmental bone defects, which was first reported in the mid-1980s (Alford et al., 2021). This procedure combines surgical techniques, bone healing mechanisms, and bone grafts, significantly promoting bone defect repair (Nauth et al., 2018). This procedure is divided into two stages. The first stage is to remove damaged tissue, implant a bone cement spacer, and install a fixation device, followed by waiting for formation of the surrounding foreign-body membrane. The secondary stage is to remove the spacer and fill the cavity with bone grafts or bone graft substitutes, followed by several months of healing (Alford et al., 2021). Though this procedure is promising, there are some problems to be solved, for example, time consumption, the lack of standard surgical details, and the shortage of evidence to supplement bioactive factors and optimize individual outcomes (Morelli et al., 2016).

### Cell-Based Therapy and Cell-Free Therapy

Cell-based and cell-free scaffolds often combine bone engineering scaffolds with cells, cytokines, nucleic acids, or extracellular vesicles, which enhance the osteoinductive and osteogenic capacity of the scaffolds (Li et al., 2019). With multilineage differentiation potential and intrinsic properties, MSCs are the most promising stem cells being applied in bone regeneration medicine to promote wound healing, osteogenesis, and inflammation modulation (Chew et al., 2019). The advancing applications of MSCs in bone regeneration have been reviewed by Shang et al. (2021). Though relevant basic and clinical translational research studies are being carried out vigorously, cell-based therapy has some limitations, such as low viability, immune rejection reaction, tumorigenesis, and microthrombosis (Watanabe et al., 2021). Alternative options are based on the secretion and paracrine signaling of MSCs, that is, cell-free therapy; for example, growth factors, cytokines, nucleic acids, and extracellular vesicles (Zhang et al., 2022; Swanson et al., 2020). The types, time, and dosage of growth factors and cytokines supplementing scaffolds are important parameters, which depend on the stages of bone healing because the biomolecules’ interaction is pretty intricate in a spatiotemporal manner (Safari et al., 2021). More efforts have been made to achieve controlled release of cytokines, which deserves further exploration. Moreover, as an important gene regulator at the post-transcriptional level, nucleic acids are supplemented to regulate gene expression and promote osteogenesis, which usually requires a carrier to transport them into the cells (Li Z. et al., 2021). In addition, extracellular vesicles possess the inherent capacity to carry biomolecules, thus mediating molecule delivery to promote regeneration (Ramis, 2020). Extracellular vesicles, including exosomes, apoptotic bodies, and microvesicles, particularly exosomes, have been reported to recapitulate the advantageous properties of stem cells and enhance the effects of bone engineering scaffolds (Pishavar et al., 2021; Qin et al., 2016). The advancing applications of exosomes and scaffolds will be elaborated in the next section.

## Applications of Bone Engineering Scaffolds With Exosomes in Bone Defects

**FIGURE 2:**
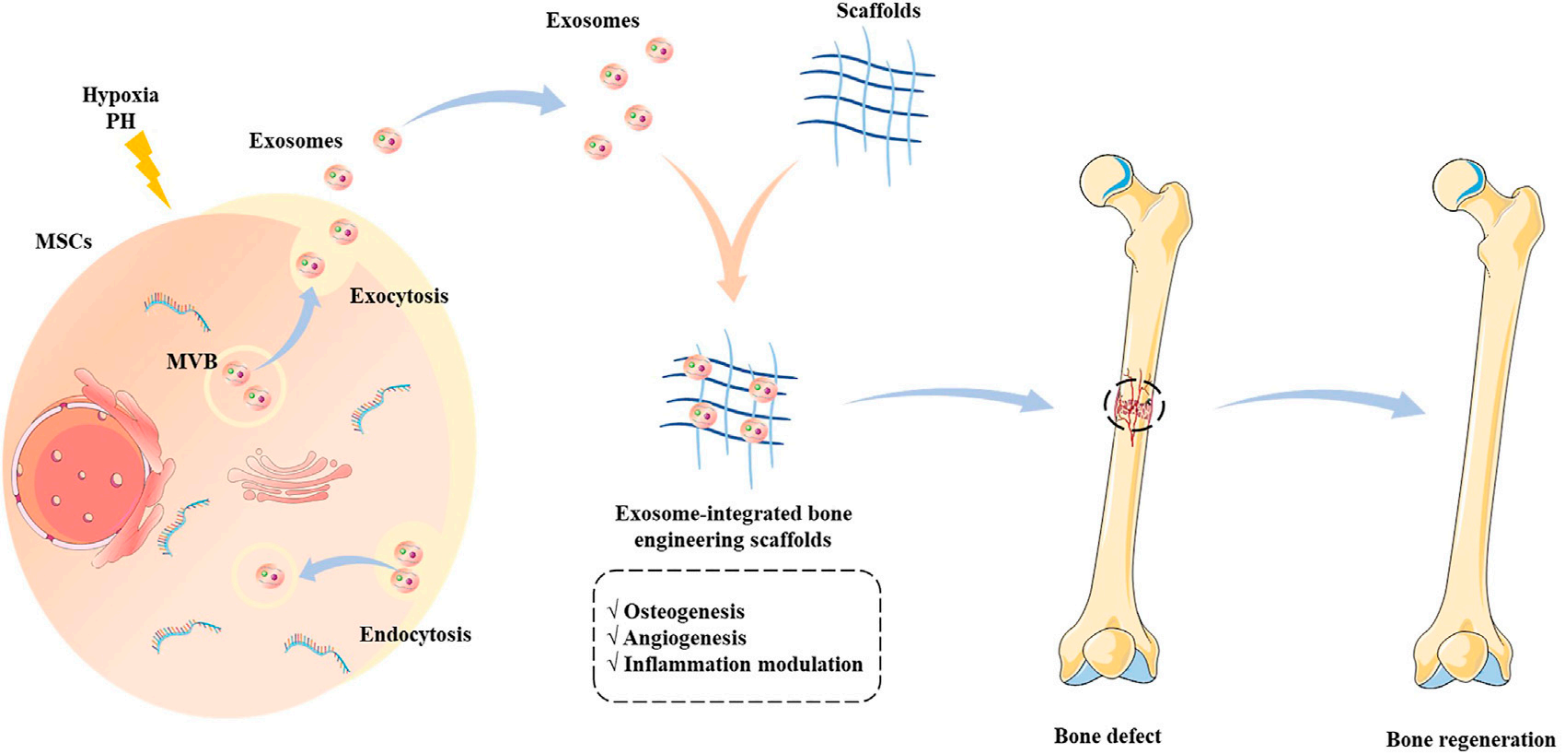
Schematic illustration of the source of exosomes and the application of exosome-integrated bone engineering scaffolds. The exosomes originate from multivesicular bodies, which engage in intercellular communications and deliver biomolecules to regulate the biological functions. The exosome-integrated bone engineering scaffolds possess the capacity for osteogenesis, angiogenesis, and inflammation modulation, which promote bone regeneration and repair bone defects.

The exosome-integrated bone engineering scaffolds synergize mechanical support and the ability of osteoconductivity, osteoinduction, and osteogenesis, which have been widely explored in bone defect animal models and achieve good therapeutic effects (Figure 2) (Wei et al., 2019). In recent years, the osteogenic property of bone engineering scaffolds has been continuously explored, and the investigation of angiogenesis has also been widely conducted (Turnbull et al., 2018).

### Exosome Overview

Exosomes, originating from multivesicular bodies, are widely found in biofluids and cell mediums, which range from 30 to 150 nm in diameter (Liu A. et al., 2021). They carry nucleic acids, proteins, lipids, and metabolites, playing key roles in intercellular communication (Lu et al., 2019). It has been reported that exosomes have diagnostic and therapeutic potential in various diseases, including bone defects (Al-Sowayan et al., 2020; Furuta et al., 2016). Compared with cell-based therapy, the sources of exosomes applied in bone defects are more widespread, which is not limited to stem cells. In addition, exosome-based therapy has several benefits, such as no immunogenicity, similar functional properties to stem cells, and no risks of tumorigenesis and engineering modification (Fan et al., 2020).

### Exosomes in Osteogenesis, Angiogenesis, and Inflammation Modulation in Bone Defect Microenvironment

Nucleic acids, proteins, lipids, and metabolites in exosomes engage in intercellular communication and impact the recipient cells to regulate biological functions (Escude et al., 2021). Relevant research studies show that exosomes are engulfed by surrounding target cells, such as osteoprogenitors, osteoblasts, endothelial cells, and immune cells, in the bone defect microenvironment, thereby widely participating in osteogenesis, angiogenesis, and inflammation modulation.

#### Exosomes and Osteogenesis

In bone regeneration, the exosomes with osteogenic potential are able to promote MSCs to increase the expression of osteogenic factors and osteogenesis-related proteins, such as RUNX2, COL1A1, OPN, and ALP (Narayanan et al., 2016). Bioactive molecules in exosomes are key mediators of osteogenesis, and several reports show that microRNA (miRNA) is an important post-transcript regulator to modulate the expression of osteogenic-related genes(Liu W.-z. et al., 2021; Yin et al., 2021). For example, the RNA-sequencing of osteogenic exosomes from human MSCs suggests that exosomes include upregulated osteogenic miRNAs (Hsa-miR-146a-5p, Hsa-miR-503-5p, Hsa-miR-483-3p, and Hsa-miR-129-5p) or downregulated anti-osteogenic miRNAs (Hsa-miR-32-5p, Hsa-miR-133a-3p, and Hsa-miR-204-5p), which activate the PI3K/Akt and MAPK signaling pathways (Zhai et al., 2020). In addition, MSC-released exosomal miR-1260a (Wu et al., 2021); miR-335 (Cao et al., 2021); miR-140 and miR-375 (Chen et al., 2019); miR-26a, miR-199a, miR-21, and miR-23a-3p (Hu et al., 2020); let-7a-5p, let-7c-5p, miR-328a-5p, and miR-31a-5p (Liu A. et al., 2021); and miR-150-5p (Jing et al., 2022) have been reported to promote osteogenesis. The exosomes promoting osteogenesis involve many pathways, such as BMP/Smad, Wnt/β-catenin, PTEN/PI3K/Akt, and Hippo signaling pathways (Cao et al., 2021; Cui et al., 2016; Hu et al., 2020; Zhang et al., 2016). There are other cell-derived exosomes promoting or inhibiting osteogenesis. For example, Li et al. (2016) showed that osteoclast-derived exosomal miR-214-3p inhibits osteogenesis and reduces bone formation. Weilner et al. (2016) reported that osteoblast-derived exosomal galectin-3 levels are positively correlated with osteoinductive potential. Qi et al. (2016) reported that human-induced pluripotent stem cell–derived MSC-released exosomes significantly promote osteogenesis and angiogenesis. Li et al. (2018) showed that human adipose stem cell–derived exosomes promote the proliferation and differentiation of MSCs. Swanson et al. (2020) found that human dental pulp stem cell–derived exosomes facilitate MSC differentiation and mineralization. Wu et al. (2020) reported that Schwann cell–derived exosomes can promote the proliferation and differentiation of MSCs. Cao et al. (2021) found that mature dendritic cell–derived exosomes enhance osteogenic differentiation of MSCs. Moreover, some studies have focused on regulating exosomes to increase their osteogenic activity, such as aptamer-functionalized exosomes, static magnetic field–treated exosomes, exosomes endowed with plasmids, genetic engineered exosomes, hypoxic environment-treated exosomes, hydrogel-assisted 3D cultured exosomes, and exosomes with fusion peptide (Li et al., 2022; Luo et al., 2019; Ma et al., 2022; Shen et al., 2022; Wu et al., 2021; Yu et al., 2022; Zha et al., 2021). In general, the treatment of exosomes increases the osteogenic ability in the bone defect microenvironment, which is beneficial for bone defect repair.

#### Exosomes and Angiogenesis

**FIGURE 3:**
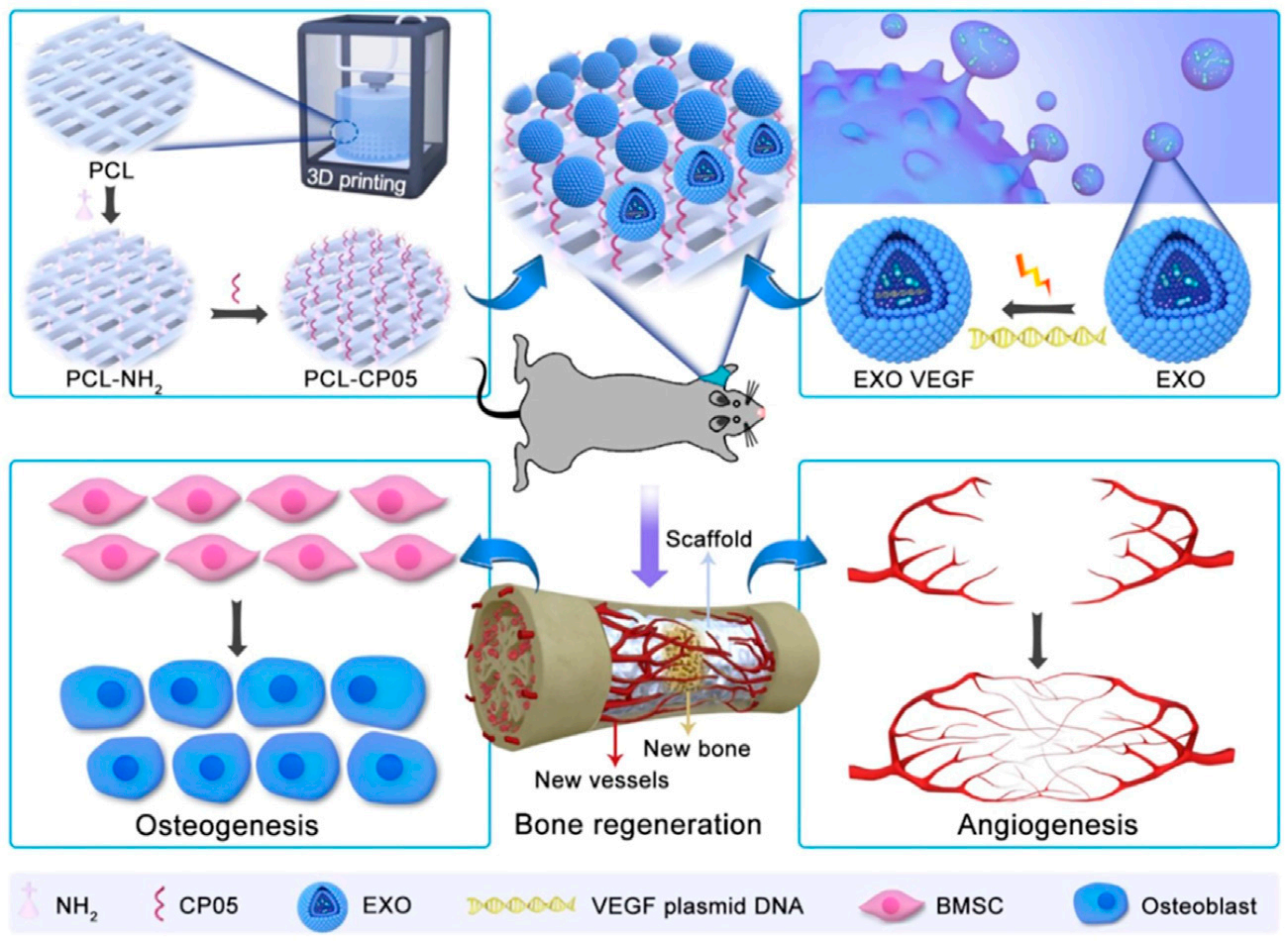
The combination of engineering exosomes endowed with VEGF plasmid with 3D-printed scaffolds promotes bone regeneration via enhancing osteogenesis and angiogenesis (Zha et al., 2021).

Adequate blood supply is an important basis for successful bone regeneration. The effects of angiogenesis mediated by exosomes have been reported, indicated by the increased expression of angiogenesis factors, tube formation, endothelial cell proliferation, and migration (Zhang J. et al., 2021). For example, Wu et al. (2022) suggested that MSC-derived exosomal miR-21 target SPRY2, promotes angiogenesis. Another report shows that miR-21 promotes angiogenesis by the miR-21/NOTCH1/DLL4 signaling axis (Zhang Y. et al., 2021). Wu et al. (2021) found that MSC-derived exosomal miR-1260a enhanced angiogenesis via the inhibition of COL4A2. Sahoo et al. (2011) showed that MSC-derived exosomes increased endothelial cell viability. Jing et al. (2022) reported that stem cells from apical papilla-derived exosomes promote angiogenesis by miR-126-5p, indicated by increased expression of VEGF and ANG-1. In addition, hypoxic condition–treated cell-derived exosomes increase the tube formation (Liu et al., 2020). Zha et al. (2021) also reported that progenitor cell–derived exosomes endowed with VEGF plasmids release the VEGF gene to promote angiogenesis (Figure 3).

#### Exosomes and Inflammation Modulation

Inflammatory cells and immune cells are important components of the bone defect microenvironment, and a moderate inflammatory response is imperative for bone defect repair (Lin et al., 2022). It has been reported that exosomes play a role in inflammation modulation. For example, MSC-derived exosomes can promote macrophage polarization toward the M2 phenotype and inhibit the inflammatory response, indicated by the reduced gene and protein expression of inflammatory cytokines, such as IL-6 and TNF-α (Guan et al., 2022; Jiang et al., 2021; Lu et al., 2021; Wang X. et al., 2020). Research shows that MSC-derived exosomes promote macrophage M2 polarization via the NF-κB pathway (Fan et al., 2021). In addition, Lin et al. (2022) showed that HUVEC-derived exosomes overexpressing PD-L1 bind to PD-1 on the T-cell surface, which suppresses the activation of T cells and promotes MSCs toward osteogenic differentiation because of the inhibition of overactive inflammation. Consequently, exosomes are important regulators of immune response and bone regeneration in the bone defect microenvironment.

To sum up, the role exosomes play in bone defect repair is multifaceted, including osteogenesis, angiogenesis, and inflammation regulation, which supplement each other. Specifically, the effects of exosomes in the bone defect microenvironment are due to 1) promotion of osteogenic differentiation of the target cells, 2) promotion of angiogenesis for providing an optimal bone regeneration niche, and 3) inflammation modulation for maintaining a moderate immune response. Thus, the exosome is a kind of promising cell-free therapeutic material to repair bone defects.

### Exosome-Integrated Bone Engineering Scaffolds *In Vivo* Bone Defects

**FIGURE 4:**
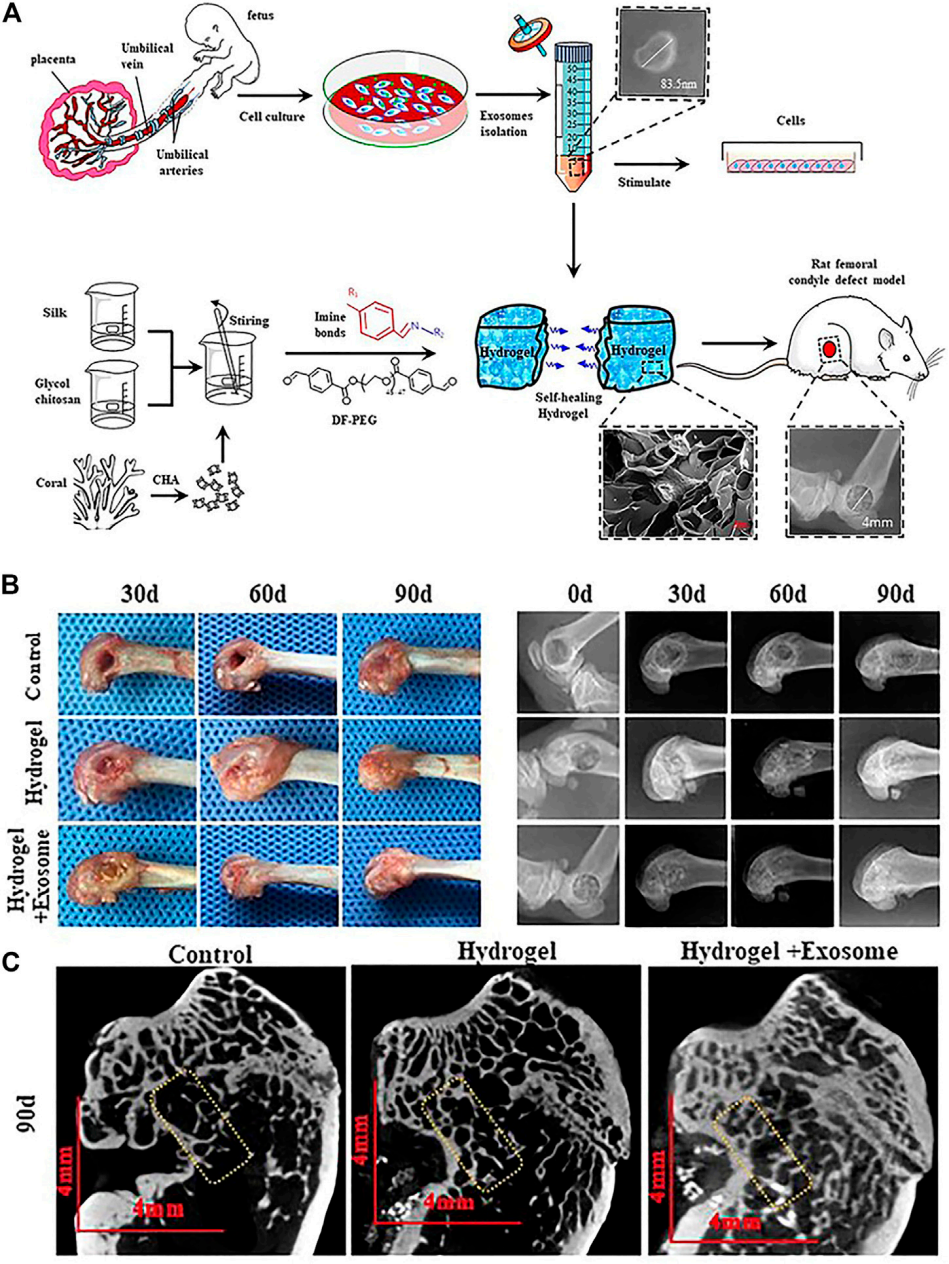
Self-healing hydrogel containing exosomes (Wang L. et al., 2020). **(A)** Schematic illustration of the isolation and characterization of exosomes and preparation of self-healing hydrogel for applying in rat femoral condyle defect. **(B)** Gross observation and X-ray evaluation of the effects of self-healing hydrogel containing exosomes. **(C)** Micro-CT evaluation of the effects of self-healing hydrogel containing exosomes.

The treatment of exosome-integrated bone engineering scaffolds provides not only mechanical support for bone defects but a suitable microenvironment for bone regeneration. The research studies on bone engineering scaffolds for bone defects primarily focus on the components, characteristics, interface modification, and the release of bioactive factors (Gandolfi et al., 2020; Nikhil and Kumar, 2022; Zhang et al., 2018). Although most of the scaffolds have been proven to possess the definite potential of osteogenesis, the supplement of exosomes can enhance their performance (Figure 4) (Tan et al., 2020; Wang et al., 2020a). Exosome-integrated bone engineering scaffolds can promote osteogenesis, angiogenesis, and inflammation modulation, which have been applied in various bone defect models, such as critical-sized mouse calvarial defects, mandibular bone defects, femoral condyle defects, and tibia defects in mice, rats, and sheep (Jia et al., 2021; Luo et al., 2019). The details of applications of exosome-integrated bone engineering scaffolds have been summarized in Table 2. In addition, exosome-integrated bone engineering scaffolds promote the recruitment and migration of resident MSCs, the activation of local potential, and the homing ability to the injured sites and newly formed bone tissue sites (Re et al., 2021). For example, Schwann cell–derived exosomes with porous titanium alloy can improve the effects of scaffolds in bone repair (Wu et al., 2020). Ma et al. (2022) reported that hydrogels combined with exosomes and fusion peptides can enhance the therapeutic effect and the retention of exosomes. Yang et al. (2020) revealed that MSC-derived exosomes with injectable hydroxyapatite-embedded *in situ* cross-linked hyaluronic acid-alginate hydrogel can significantly enhance bone regeneration and retain the exosomes at the defect sites. Although bone engineering scaffolds with exosomes show great potential, there are still some questions to be answered. First, the contents and their functions in exosomes have not yet been illustrated completely, and the effectors of exosomes of different parent sources for bone defect repair are different. Second, the scaffold materials with better osteoconductivity, osteoinduction, osteogenesis, and mechanical support need to be improved. Lastly, the strategies of engineering exosomes, interface modifications, and controlled release in a spatiotemporal manner need to be designed and optimized. To sum up, the progress in materials and engineering technology drives the bone graft substitutes to solve the clinical problems, pointing out the direction of future research studies.

**TABLE 2:** Summary of the applications of exosome-integrated bone engineering scaffolds in bone defects.

Source of Exosomes	Isolation method	Scaffold	*In vivo* model	Effect of exosome-integrated bone engineering scaffolds	References
Human umbilical cord MSCs	Ultracentrifugation	Coralline hydroxyapatite (CHA)/silk fibroin (SF)/glycol chitosan (GCS)/difunctionalized polyethylene glycol (DF-PEG) self-healing hydrogel	Femoral condyle defect	Pro-bone regeneration and pro-angiogenic activities *in vitro* and *in vivo*	Wang et al. (2020a)
Human umbilical cord MSCs	Ultracentrifugation	Injectable hydroxyapatite-embedded *in situ* cross-linked hyaluronic acid-alginate (HA-ALG) hydrogel	Calvarial defect	Pro-bone regeneration activities *in vitro* and *in vivo*	Yang et al. (2020)
Bone marrow stem cells	Ultracentrifugation	Tannic acid–modified sulfonated polyetheretherketone	Femoral condyle defect	Inflammation modulation and pro-bone regeneration activities *in vitro* and *in vivo*	Fan et al. (2021)
Human adipose-derived stem cells	Ultracentrifugation	Polydopamine-coating PLGA (PLGA/pDA) scaffolds	Calvarial defect	Pro-bone regeneration activities *in vitro* and *in vivo*	Li et al. (2018)
MSCs derived from human-induced pluripotent stem cells	Ultracentrifugation	Tricalcium phosphate scaffolds	Calvarial defect	Pro-bone regeneration and pro-angiogenic activities *in vitro* and *in vivo*	Qi et al. (2016)
MSCs and preosteoblasts	ExoEasy kit	Calcium sulfate-nano-hydroxyapatite nanocement bone filler	Tibia defect	Pro-bone regeneration activities *in vitro* and *in vivo*	Teotia et al. (2021)
MSCs	Ultracentrifugation	Mesoporous bioactive glass	Calvarial defect	Pro-bone regeneration activities *in vitro* and *in vivo*	Liu et al. (2021a)
Schwann cells	Ultracentrifugation	Porous Ti6Al4V scaffolds	Femoral condyle defect	Pro-bone regeneration activities *in vitro* and *in vivo*	Wu et al. (2020)
Human dental pulp stem cells	Ultracentrifugation	Poly(lactic-co-glycolic acid) and poly(ethylene glycol) triblock copolymer microspheres	Calvarial defect	Pro-bone regeneration activities *in vitro* and *in vivo*	Swanson et al. (2020)
MSCs	ExoQuick-TC kit	3D-printed titanium alloy scaffolds	Radial bone defect	Pro-bone regeneration activities *in vitro* and *in vivo*	Zhai et al. (2020)
Chondrogenic progenitor cell line, ATDC5	Ultracentrifugation	3D-printed polycaprolactone scaffolds	Radial bone defect	Pro-bone regeneration and pro-angiogenic activities *in vitro* and *in vivo*	Zha et al. (2021)
Human adipose mesenchymal stem cells	Ultracentrifugation	Hydrogel	Calvarial defect	Pro-bone regeneration activities *in vitro* and *in vivo*	Chen et al. (2019)
Bone marrow stem cells	Ultracentrifugation	Injectable thermo-sensitive hydrogels	Calvarial defect	Pro-bone regeneration activities *in vitro* and *in vivo*	Ma et al. (2022)
Umbilical MSCs	Ultracentrifugation	Hyaluronic acid hydrogel	Calvarial defect	Pro-bone regeneration and pro-angiogenic activities *in vitro* and *in vivo*	Zhang et al. (2021d)
Mature dendritic cells	Ultracentrifugation	Carboxymethyl cellulose-based hydrogel	Femoral bone defect	Pro-bone regeneration activities *in vitro* and *in vivo*	Cao et al. (2021)
MSCs derived from human-induced pluripotent stem cells	Ultracentrifugation	Tricalcium phosphate scaffolds	Calvarial defect	Pro-bone regeneration activities *in vitro* and *in vivo*	Zhang et al. (2016)

## Conclusion

The treatment of bone defects is an intractable clinical problem and has attracted great attention around the world. In this review, the current treatments of bone defects and applications of bone engineering scaffolds with exosomes in bone defects are summarized. In addition, the bone defect microenvironment and bone healing mechanism are discussed. In bone defect repair, the supplement of exosomes enhances the effects of bone engineering scaffolds, in which miRNA is one of the important regulators. With the recognition of exosome contents, future patterns of specific miRNA or bioactive molecules with carriers will promote bone regeneration more precisely. Thus, a big step forward would be taken toward the successful treatment of bone defects, delayed union, and non-union.
